# Diagnosis and management of leukocytoclastic vasculitis

**DOI:** 10.1007/s11739-021-02688-x

**Published:** 2021-03-13

**Authors:** Paolo Fraticelli, Devis Benfaremo, Armando Gabrielli

**Affiliations:** Dipartimento Di Scienze Cliniche E Molecolari, Clinica Medica, Università Politecnica Delle Marche, Via Tronto 10/A, 60127 Ancona, Italy

**Keywords:** Leukocytoclastic vasculitis, Small vessel vasculitis, Cryoglobulinemic vasculitis, IgA vasculitis, Hypocomplementemic urticarial vasculitis

## Abstract

Leukocytoclastic vasculitis (LCV) is a histopathologic description of a common form of small vessel vasculitis (SVV), that can be found in various types of vasculitis affecting the skin and internal organs. The leading clinical presentation of LCV is palpable purpura and the diagnosis relies on histopathological examination, in which the inflammatory infiltrate is composed of neutrophils with fibrinoid necrosis and disintegration of nuclei into fragments (“leukocytoclasia”). Several medications can cause LCV, as well as infections, or malignancy. Among systemic diseases, the most frequently associated with LCV are ANCA-associated vasculitides, connective tissue diseases, cryoglobulinemic vasculitis, IgA vasculitis (formerly known as Henoch–Schonlein purpura) and hypocomplementemic urticarial vasculitis (HUV). When LCV is suspected, an extensive workout is usually necessary to determine whether the process is skin-limited, or expression of a systemic vasculitis or disease. A comprehensive history and detailed physical examination must be performed; platelet count, renal function and urinalysis, serological tests for hepatitis B and C viruses, autoantibodies (anti-nuclear antibodies and anti-neutrophil cytoplasmic antibodies), complement fractions and IgA staining in biopsy specimens are part of the usual workout of LCV. The treatment is mainly focused on symptom management, based on rest (avoiding standing or walking), low dose corticosteroids, colchicine or different unproven therapies, if skin-limited. When a medication is the cause, the prognosis is favorable and the discontinuation of the culprit drug is usually resolutive. Conversely, when a systemic vasculitis is the cause of LCV, higher doses of corticosteroids or immunosuppressive agents are required, according to the severity of organ involvement and the underlying associated disease.

## Introduction

The term leukocytoclastic vasculitis (LCV) refers to an histopathologic description of a common form of small vessel vasculitis (SVV), involving arterioles, capillaries and postcapillary venules, in which the inflammatory infiltrate is composed of neutrophils with fibrinoid necrosis and disintegration of nuclei into fragments (“leukocytoclasia”) [1].

The microscopic changes of LCV may be found in various types of vasculitis affecting the skin and internal organs, although the name LCV more typically refers to small-vessel vasculitis of the skin. Moreover, since the terms “cutaneous LCV,” “cutaneous small-vessel vasculitis” and “cutaneous leukocytoclastic angiitis” are used interchangeably, there is often considerable confusion.

In the 2012 revised International Chapel Hill Consensus Conference (CHCC) nomenclature of vasculitides [2], LCV has been classified among single organ vasculitides, due to the prevalent involvement of the skin. More recently, a Dermatologic Addendum to CHCC 2012 [3] updated the former classification, recognizing that cutaneous SVV could be (1) a skin component of systemic vasculitis; (2) a skin-limited or skin-dominant expression or variant of a systemic vasculitis; (3) a single-organ vasculitis that differs with regard to clinical, laboratory, and pathologic features from recognized systemic vasculitides. Accordingly, histologically LCV may be found in numerous conditions, both skin-limited and systemic diseases.

In this narrative review, we will outline the current approach to the diagnosis and management of cutaneous and systemic LCV.

### Histopathological definition

LCV is a term that describes the histopathological entity characterized by: (1) evidence of neutrophilic infiltration within and around the vessel wall with signs of leukocytoclasia (disintegration of neutrophil nuclei into fragments or nuclear dust); (2) fibrinoid necrosis (fibrin deposition within and around the vessel walls); (3) signs of damage of the vessel wall and surrounding tissue (e.g., extravasated red blood cells, damaged endothelial cells) [4].

Although these features are usually pathognomonic, the histopathological diagnosis of LCV may be challenging, because abnormalities tend to evolve over time. In fact, before the development of full-blown LCV changes, the specimen may only show focal damage of capillary blood vessels with a mild granulocytic infiltrate with or without foci of leukocytoclasia. Otherwise, in older lesions the inflammatory infiltrate may be richer in lymphocytes rather than neutrophils.

Sometimes IgA or IgM/IgG immune complexes can be found in direct immunofluorescence studies suggesting specific forms of LCV. However, it should be remembered that the histopathological pattern is not specific for any particular entity; therefore, the presence of LCV must be related to clinical features before making a definitive diagnosis.

### Epidemiology

Although there is considerable uncertainty due to the variability of its definition, the incidence of cutaneous LCV ranges from 15 to 38 cases per million/year, whereas the prevalence from 2.7 to 29.7 per million [5–7]. A recent population wide study estimated an incidence of 4.5 per 100.000 person-years (95% CI, 3.5–5.4) for biopsy-proven LCV in the United States [8].

Cutaneous LCV appears to affect both sexes equally, as well as patients of all ages, although some studies noted a slight predilection for male sex and older age. Notably, in children, IgA vasculitis is much more common than non-IgA mediated vasculitis, whereas in adults LCV is more commonly associated with an underlying systemic vasculitis, connective tissue disease, or malignancy [7–9].

### Etiology and pathogenesis

According to the revised CHCC, histological LCV can be found in: (1) ANCA-associated vasculitis (AAV), (2) immune complex vasculitis, such as Cryoglobulinemic Vasculitis (CV), IgA-Vasculitis (Henoch–Schonlein purpura, HSP), Hypocomplementemic Urticarial Vasculitis (anti-C1q vasculitis, HUV) and IgM/IgG immune complex vasculitis (formerly known as Hypersensitivity Vasculitis), (3) vasculitis associated with systemic diseases (e.g., rheumatoid arthritis, systemic lupus erythematosus and sarcoidosis) and (4) in the so-called vasculitis associated with probable etiology (e.g., related to infections, medications, sepsis or cancer) (Table 1).

**Table 1:** Classification and causes of leukocytoclastic vasculitis

CHCC 2012 category	Causes and/or associated diseases	CHCC 2012 definition	Histopathology
ANCA-associated vasculitis	Granulomatosis with polyangiitis (GPA) Microscopic polyangiitis (MPA) Eosinophilic granulomatosis with polyangiitis (EGPA)	Necrotizing vasculitis with few or no immune deposits, predominantly affecting small vessels (i.e., capillaries, venules, arterioles, and small arteries); associated with ANCAs	Vasculitis of small-to-medium vessels in the skin, often with leukocytoclasia with or without granulomatous inflammation
Immune complex vasculitis		Vasculitis with moderate-to-marked vessel wall deposits of immunoglobulin and/or complement components, predominantly affecting small vessels (i.e., capillaries, venules, arterioles, and small arteries)	LCV of small vessels (mostly postcapillary venules, occasionally small veins or arterioles)
	Cryoglobulinemic Vasculitis (CV)	Vasculitis with cryoglobulin immune deposits affecting small vessels (predominantly capillaries, venules, or arterioles); associated with serum cryoglobulins	LCV of small vessels (postcapillary venules, small veins, or arterioles); associated with serum cryoglobulins (usually type II and type III)
	IgA-Vasculitis (Henoch–Schonlein purpura, HSP)	Vasculitis with IgA1-dominant immune deposits, affecting small vessels (predominantly capillaries, venules, or arterioles)	Leukocytoclastic IgA1-dominant vasculitis of mostly postcapillary venules and also veins or arterioles in the skin, with vascular IgA deposits
	Hypocomplementemic Urticarial Vasculitis (anti-C1q vasculitis, HUV)	Vasculitis accompanied by urticaria and hypocomplementemia, affecting small vessels (i.e., capillaries, venules, or arterioles) and associated with anti-C1q antibodies; common forms include glomerulonephritis, arthritis, obstructive pulmonary disease, and ocular inflammation	Cutaneous LCV of mostly postcapillary venules with vascular deposits of immunoglobulins, and manifesting with lasting urticarial lesions; anti C1q antibodies may be present
	IgM/IgG immune complex vasculitis*	Vasculitis with IgM and/or IgG-dominant immune deposits, affecting small vessels (predominantly capillaries, venules, or arterioles)	Leukocytoclastic IgM and/or IgG-dominant vasculitis of mostly postcapillary venules and also veins or arterioles in the skin, with vascular deposits
Vasculitis associated with systemic diseases	Rheumatoid arthritis Systemic lupus erythematosus Sjögren syndrome Sarcoidosis	Vasculitis that is associated with and maybe secondary to (caused by) a systemic disease (e.g., rheumatoid vasculitis, SLE, sarcoid vasculitis, etc.); the name (diagnosis) should have a prefix term specifying the systemic disease (e.g., rheumatoid vasculitis, lupus vasculitis, etc.)	Cutaneous LCV as a component of systemic vasculitis; the type of cutaneous vasculitis (small vessel or medium vessel vasculitis) varies depending on the underlying systemic disease
Vasculitis associated with probable etiology	Drugs Infection Sepsis Neoplasms	Vasculitis that is associated with a probable specific etiology, e.g., drug, infection, sepsis, neoplasm, etc	Cutaneous LCV as a component of systemic vasculitis that is associated with a probable specific etiology, e.g., drug, sepsis, etc

^*^Provisional category not included in the CHCC 2012

An underlying cause or a systemic involvement is usually found in around half of the cases of LCV, whereas the other 50% is split between a single-organ cutaneous SVV, usually drug or infection induced, or an idiopathic/unclassifiable LCV [10–14].

The cutaneous component of systemic AAV may present with LCV of dermal postcapillary venules, sometimes extending into arterioles or small veins, and clinically manifesting as hemorrhagic papules or macules, sometimes nodules. On immunofluorescence immune deposits are usually absent. The involvement of larger vessels manifests with livedo reticularis, ulcers and/or nodules [15].

Immune complex vasculitis is characterized by moderate-to-marked vessel wall deposits of immunoglobulin and/or complement components, predominantly affecting small vessels (i.e., capillaries, venules, arterioles, and small arteries) [4], [16]. Systemic variants of immune complex vasculitis include CV, which is associated with serum cryoglobulins (usually type II and type III), HSP, a vasculitis with IgA1-dominant immune deposits, affecting small vessels, and HUV or anti-C1q vasculitis, which is accompanied by urticaria and hypocomplementemia. All these systemic conditions may also be limited to the skin.

Henoch–Schönlein purpura (HSP) is the most common form of vasculitis occurring in childhood, affecting 10–20 children per 1,00,000 per year. More than 90% of patients are under 10 years of age, with a mean age of 6 years [17]. Its clinical presentation includes the classic tetrad of palpable purpura, joint pain, gastrointestinal complaints, and renal involvement. The skin-limited form of IgA vasculitis is much more common in adults than children.

Urticarial vasculitis (UV) can be divided into two groups according to complement levels, normocomplementemic UV and hypocomplementemic UV (HUV), the latter also being called anti-C1q vasculitis.

While most of the normocomplementemic UV are idiopathic, HUV may be associated with systemic diseases, such as SLE, primary Sjögren’s syndrome, and monoclonal gammopathy as well as with hematologic disorders and drug hypersensitivity.

Cryoglobulinemic syndrome or cryoglobulinemic vasculitis (CV) is a small vessel vasculitis involving the skin, the joints, the peripheral nervous system and the kidneys, associated with cryoglobulinemia. Cryoglobulins are circulating immunoglobulins (Ig) that precipitate with cold temperature and are able to form immune-complexes, mainly rheumatoid factor (RF)-like antibodies, almost invariably an IgM against an IgG which reversibly precipitate at a temperature below 37 °C.

Before the discovery of hepatitis C virus (HCV), most of CV cases were labeled as idiopathic. Nowadays, chronic HCV infection is considered the main cause of CV, accounting for 80–85% of cases in several studies [18]. Causes of HCV-unrelated CV, accounting for 9–15% of all CV cases, include HBV and HIV infection or CTDs like Sjogren’s syndrome, SLE, rheumatoid arthritis and other autoimmune disorders, monoclonal gammopathy or hematological neoplasms [19].

Cutaneous IgM/IgG immune complex vasculitis has been provisionally categorized under the single-organ vasculitides affecting the skin and includes cases of LCV with IgG/IgM deposits that do not belong to one of the other defined immune complex vasculitides. It is an LCV of postcapillary venules that is clinically almost indistinguishable from IgA vasculitis, but on immunofluorescence microscopy IgM and/or IgG are usually seen instead of IgA [20], [21], [22]. Most cases of LCV labeled as “idiopathic” can probably fall into this umbrella category of non-IgA immune complex vasculitis [7], [8].

Cutaneous LCV may be secondary to systemic conditions, such as connective tissue diseases. Rheumatoid vasculitis occurs in patients with high titers of rheumatoid factor, longstanding disease, and who often have a severe erosive rheumatoid arthritis. In the skin, it ranges from (often IgG/IgM-positive, but also IgA-positive) LCV of postcapillary venules [21] to arteritis at the dermal-subcutaneous junction or in the panniculus [23]. A more frequent involvement of vessels larger than postcapillary venules distinguishes it from proper IgA or IgG/IgM vasculitis and results in a more varied clinical presentation, including cutaneous ulcers, digital gangrene, or nailfold infarction.

A similarly heterogeneous presentation of vasculitides may occur in systemic lupus erythematous (SLE), Sjogren’s syndrome and, more rarely, in dermatomyositis or systemic sclerosis. Cutaneous vasculitis in patients with SLE may present as hypocomplementemic vasculitis or as an immune complex vasculitis, both of which are restricted primarily to postcapillary venules [24], [25]. Hypergammaglobulinemic purpura is characterized by hypergammaglobulinemia, recurring purpura, elevated erythrocyte sedimentation rate, and the presence of rheumatoid factor. Its association with autoimmune diseases, especially Sjogren's syndrome and SLE, has been reported, although it is considered idiopathic when there is no other associated disease [26].

Finally, the occurrence of LCV in the course of sarcoidosis has been described, although it is a rare manifestation [27], [28].

Identifiable probable etiologies for vasculitis include drugs, infections, sepsis, and neoplasms.

Drug-induced vasculitis may be skin-limited or systemic. In the latter case, arthritis, GI system or kidney involvement and fever are the most frequently reported manifestations [29]. Antibiotics, mainly beta-lactams, and non-steroidal anti-inflammatory drugs (NSAIDs) are the most commonly involved drugs [30], although several other compounds have been recently reported [31]. Among them, the agents most frequently implicated in LCV were tumor necrosis factor (TNF) inhibitors [32], rituximab [33], tocilizumab [34], statins [35], and immune checkpoint inhibitors (ICI) [36].

In the largest study about this topic, TNF inhibitors were found to induce mostly cutaneous SVV (manifesting with palpable purpura), although systemic vasculitis was also frequently observed [32]. Importantly, no patient experienced a recurrence of vasculitis after therapy discontinuation.

The first three cases of cutaneous LCV induced by ICI therapy have been recently described [36]. Two patients received nivolumab and one patient pembrolizumab. All three patients received combination therapy with hydroxychloroquine and corticosteroids, with complete clinical remission in few days.

A relevant criterion for suspecting skin-limited, drug-induced vasculitis is the temporal association of onset with intake and the eventual reversibility with discontinuation of the drug. Therefore, whenever it applies, the definition of “drug-induced” takes on a favorable meaning, since the vasculitis usually remits upon drug discontinuation and will not recur unless it is reintroduced.

Among infectious causes, upper respiratory infections (such as beta-hemolytic Streptococcus group A) are commonly implicated in cutaneous LCV; however, a variety of infectious triggers have been reported (Coxiella, Parvovirus, Rubeola and mumps sometimes may induce a purpuric rash subsequent to a LCV) [37], [38], [39]. Vasculitis tends to occur 7–10 days after exposure to a drug or infectious trigger.

Chronic infection with Hepatitis C and Hepatitis B viruses is associated with two particularly severe forms of systemic vasculitis involving small and medium vessels: Cryoglobulinemic vasculitis and Polyarteritis nodosa, respectively.

Patients with paraneoplastic vasculitis are usually older, more frequently have constitutional symptoms and most commonly have un underlying hematologic malignancy [40], [41].

### Clinical manifestations

**Fig. 1:**
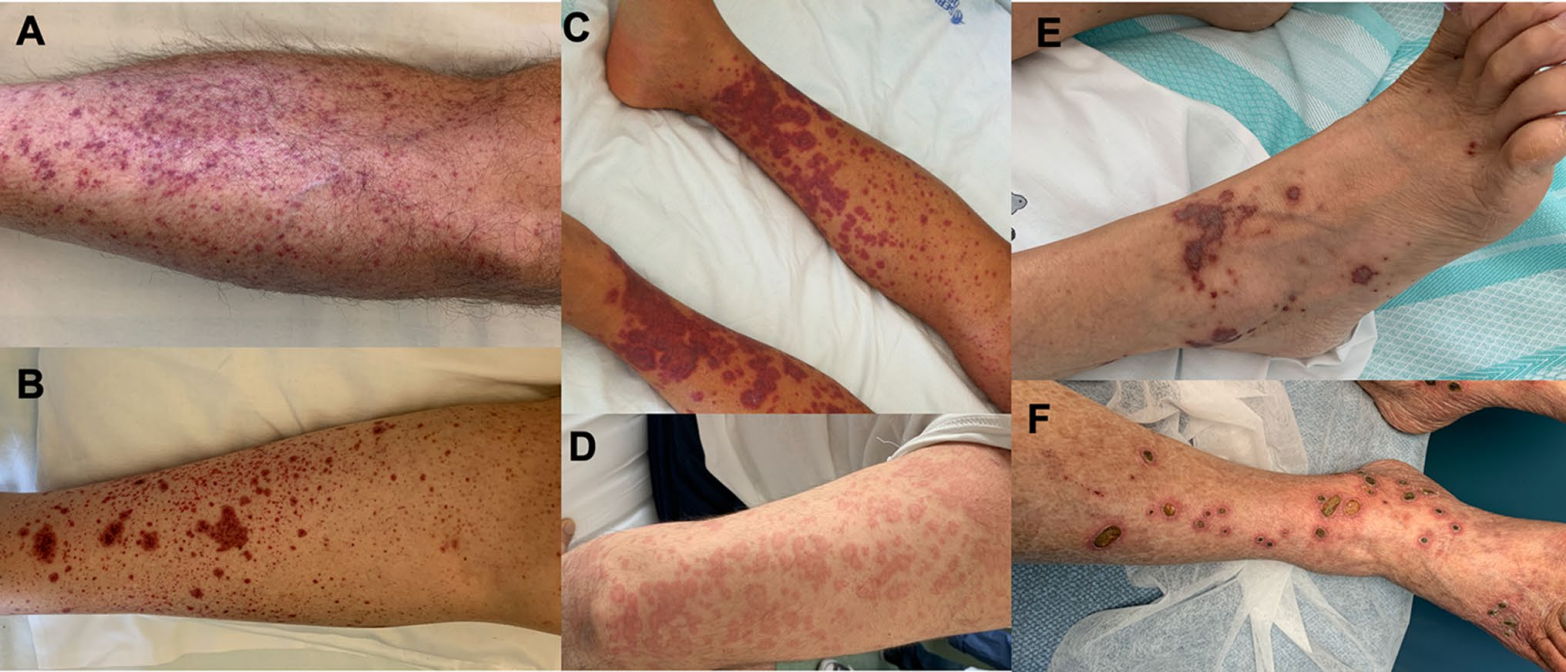
Clinical presentation of LCV. Most frequent cutaneous lesions are petechiae (panel A), purpura (panel B), confluent purpura (panel C), urticarial wheals (panel D), bullous-hemorrhagic purpura (panel E) and deep-skin ulcers and nodules (panel F)

The leading clinical presentation of LCV is palpable purpura (Fig. 1a, b). The papules usually develop over few hours, simultaneously or sequentially, and involve primarily the lower legs, but dependent areas, such as the back in the hospitalized patient, may also be affected. Frequently the lesions tend to merge with confluent aspects that may cover wide skin areas (Fig. 1c). The lesions resolve over 2–3 weeks and slowly fade away, leaving behind post-inflammatory hyperpigmentation.

Patients affected by LCV may be completely asymptomatic or complain of burning, itching, or pain in the involved skin.

In addition to typical palpable purpura, LCV may also present with hive-like papules and plaques that resemble urticarial wheals (Fig. 1d). However, unlike true urticaria these lesions tend to persist longer than 24 h, to burn rather than itch, and to leave behind bruise-like, ecchymotic marks on resolution. The presence of erythematous plaques, livedo reticularis, bullous hemorrhagic lesions (Fig. 1e) or deep skin ulcers and nodules (Fig. 1f), reflects the involvement of medium-sized arteries and should raise suspicion of a medium-vessel vasculitis.

A careful medical history and thorough physical examination are essential to stratify patients based on the likelihood of underlying systemic involvement or disease. The medical history should focus on the symptoms and signs of systemic vasculitis, such as fever, weight loss, and other constitutional symptoms; arthralgia or arthritis; myalgia; abdominal pain, melena or hematochezia; cough, hemoptysis, or dyspnoea; hematuria; sinusitis or rhinitis; and paresthesia, weakness or foot drop.

If one or more of these symptoms is present, a targeted workup should be performed, in order to identify severe extracutaneous manifestations of systemic vasculitis. Additionally, potential triggers, including preceding infections, ingestion of drugs, and comorbid medical conditions, should also be investigated and recorded.

Finally, the pattern of disease evolution is often useful to carry out a differential diagnosis. Briefly: (1) a single simultaneous course of vasculitis is more frequently due to a drug or infection, (2) recurrent bouts of purpuric rash with periods of remission are suggestive of HSP, CV or Immune-complex vasculitis and (3) chronic persistent occurrence of LCV lesions may be observed in patients with systemic SVV, connective tissue diseases or paraneoplastic syndromes (hematological or solid malignancies).

### Diagnosis

**Fig. 2:**
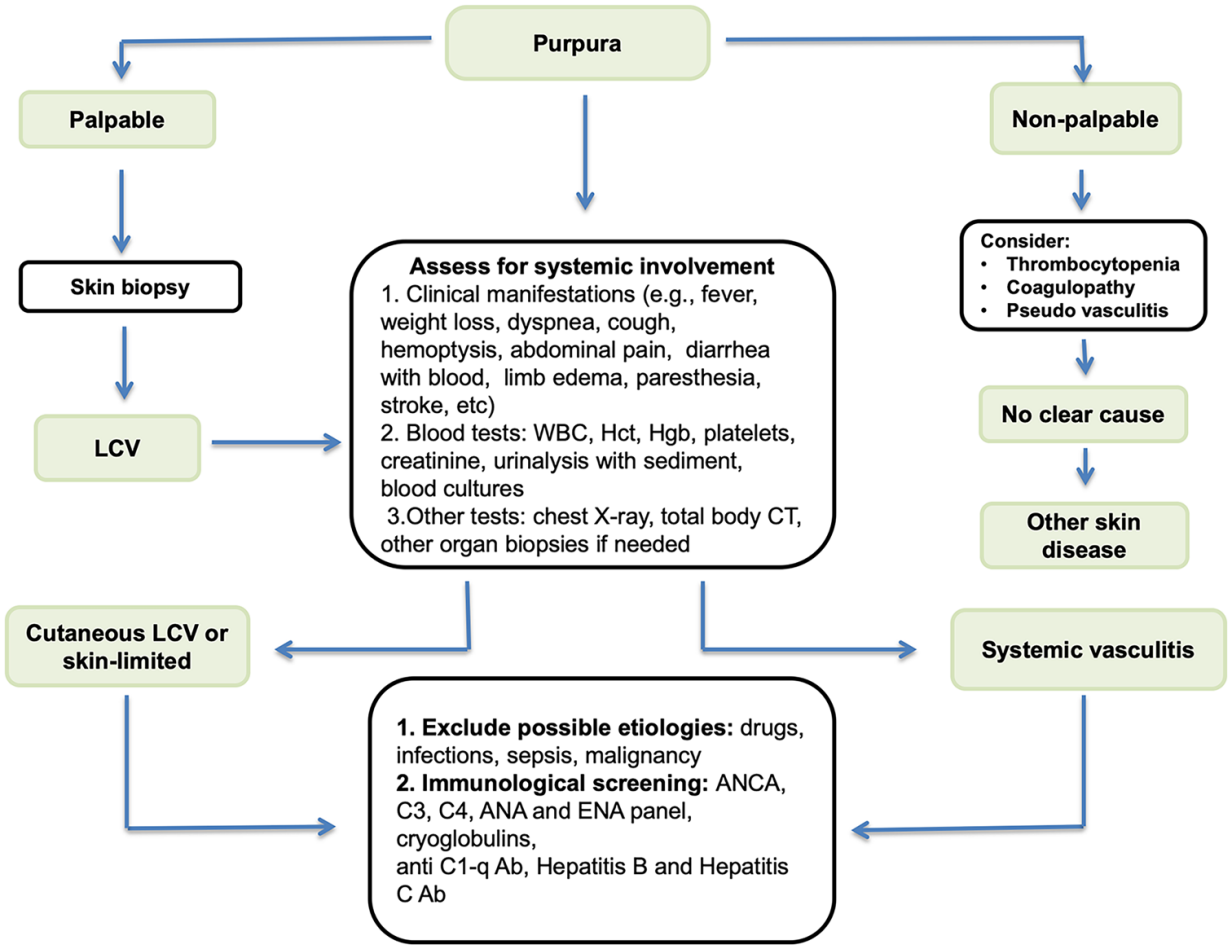
Diagnostic algorithm of leukocytoclastic vasculitis Abbreviations *WBC* white blood cells, *Hct* hematocrit, *Hgb* hemoglobin, *CT* computed tomography. *ANCA* anti-neutrophil cytoplasmic antibodies; *ANA* antinuclear antibodies; *ENA* extractable nuclear antibodies

Although history and physical examination are sometimes sufficient to establish a working diagnosis, systematic laboratory workup is usually warranted to perform the differential diagnosis. Since there is no standardized protocol, the workup should be guided by the clinical presentation (Fig. 2).

When the presentation is clear (e.g., there is a probable drug or infectious trigger), and there is no sign of systemic disease, obtaining a complete blood count, chemistry, and urinalysis could be sufficient.

In all the other cases, a reasonable workup should include infectious serologies (e.g., hepatitis B and C, human immunodeficiency virus), serum protein electrophoresis, immunoglobulins (IgG, IgA, IgM), antinuclear antibody panel and rheumatoid factor, serum C3 and C4 complement levels, ANCAs and cryoglobulins.

Circulating cryoglobulins can be detected as protein precipitates in patients’ serum maintained at 4 °C during at least 7 days, which dissolve when heated at 37 °C. Cryoprecipitate can be classified into three types, according to Brouet et al.: type I, when composed by a monoclonal immunoglobulin (IgG or IgM), type II, when a monoclonal antibody (IgM) reacts with polyclonal immunoglobulins (IgG), and type III, when both immunoglobulins (IgG or IgM) are polyclonal [42].

Whenever necessary, further targeted workup, such as imaging studies, should be considered based on the cues in the history and physical examination. Finally, given their non-specificity, the presence of leukocytosis, raised C-reactive protein levels or arthralgia are not sufficient evidence of systemic vasculitis.

Skin biopsy is of paramount importance and should be performed whenever possible to confirm the diagnosis of LCV. If cutaneous vasculitis is suspected, it is essential that the specimen sent to the pathologist includes not only the superficial dermis, but also the deep layers of the skin, such as hypodermis. In most cases, a punch biopsy should be sufficient to sample the entire dermis, whereas a wedge biopsy should be considered to sample medium-sized vessels and rule out the presence of a medium or small-to-medium vessel vasculitis.

Due to the natural history of vasculitic bouts, the timing and location of the biopsy are critical in obtaining a diagnostic sample. Ideally, lesions should be sampled within 48 h of their occurrence but not too early, because typical LCV results can be missed. Additionally, whenever possible, biopsy should be performed before starting steroid therapy, when indicated, as immunofluorescence may be easily biased.

**Fig. 3:**
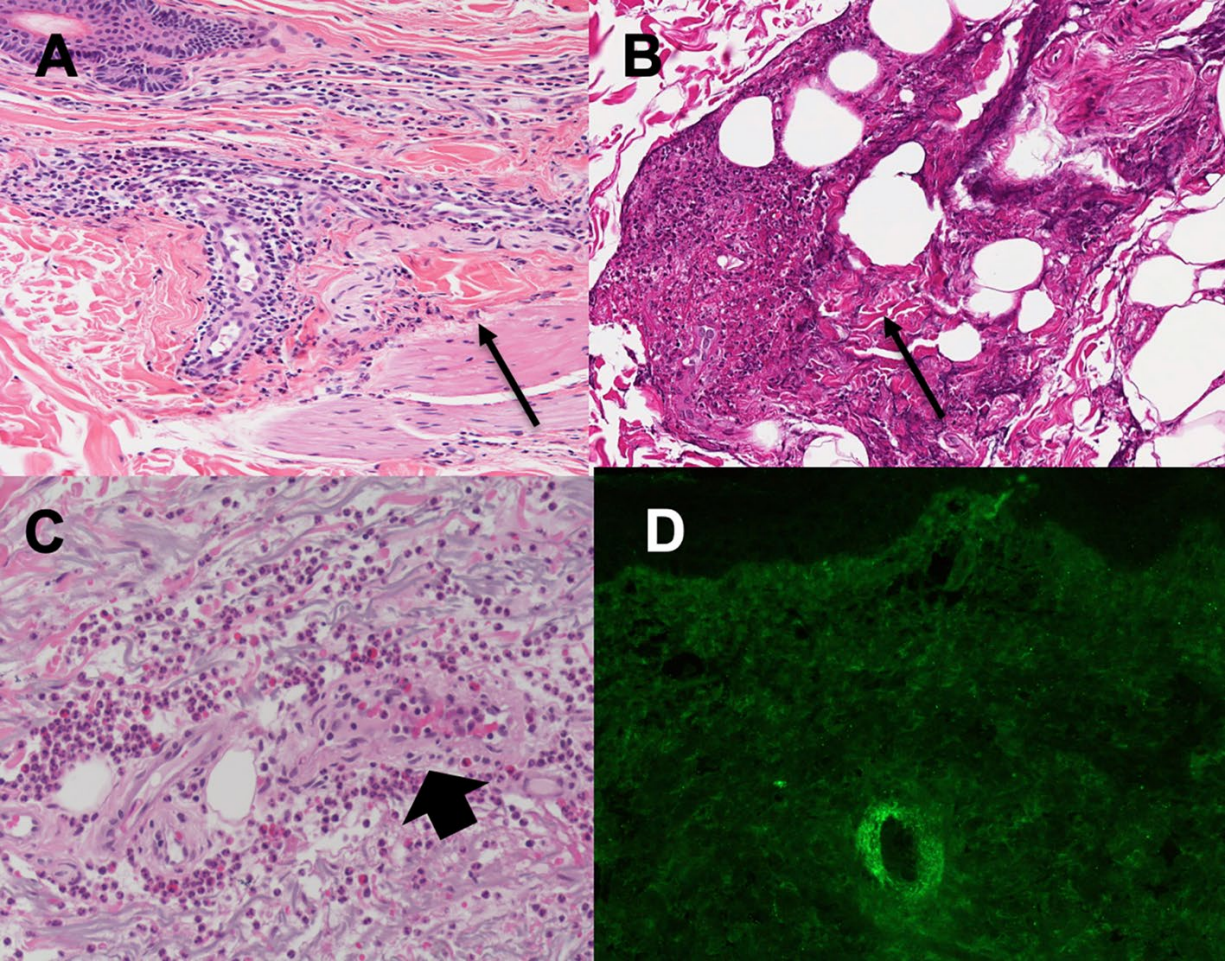
Histopathological findings in LCV. Skin biopsy with evident perivascular neutrophilic infiltrate in the dermis with fibrinoid deposits (arrow) (**a**). Fibrinoid necrosis (arrow) of deep large arterioles in the subdermal fat panniculus (**b**). Eosinophils rich mixed to neutrophilic perivascular infiltrate (arrowhead) of an urticarial vasculitis (**c**). IFI staining for IgA deposits surrounding a cutaneous vessel (**d**)

As already mentioned, the prototypical findings of leukocytoclastic vasculitis include a neutrophilic infiltrate of superficial and mid dermal small blood vessels, granulocytic debris and nuclear dust (leukocytoclasia), fibrinoid necrosis and disruption of vessel walls, and extravasation of red blood cells into the surrounding tissue (Fig. 3). A mixed inflammatory infiltrate may also be present, particularly in older lesions (Fig. 3a, b). The presence of tissue eosinophilia suggests the vasculitis may be drug-induced or may be the expression of urticarial vasculitis (Fig. 3c.

Whenever possible, a second biopsy, or part of the specimen, not formalin fixed paraffin embedded, should be used for direct immunofluorescence studies. Beyond its diagnostic value, detection of immune complex deposition may also have prognostic relevance. In fact, the presence of IgA deposits is diagnostic for HSP syndrome [43] (Fig. 3d). The deposition of C3 and/or IgG at the dermo-epidermal junction is suggestive for HUV and systemic lupus erythematosus, while IgM deposition is suggestive of autoimmune or inflammatory diseases [43]. Because the subsequent inflammatory cascade destroys the immune complexes, older lesions may be falsely negative.

LCV may be difficult to distinguish from other causes of purpura (Table 2). Vasculitic purpura is most frequently a palpable purpura, due to infiltration and inflammation of the superficial layers of the skin. Non-palpable purpura, on the other hand, is usually due to non-inflammatory vessel wall abnormalities with increased capillary fragility (scurvy, Ehlers–Danlos Syndrome, amyloidosis, steroid purpura, solar purpura, exercise purpura) or hematological or clotting disorders (e.g., thrombocytopenia, clotting defects). Pseudovasculitis, presenting both as palpable or nonpalpable purpura, include several potentially severe diseases, such as infectious emboli (e.g., from endocarditis), acute meningococcemia, disseminated gonococcal infection, Rocky mountain spotted fever, disseminated intravascular coagulation, monoclonal paraproteinemias or Waldenstrom’s disease, thrombotic thrombocytopenic purpura (Moskowitz’s disease), emboli due to cardiac myxoma and cholesterol emboli.

**Table 2:** Differential diagnosis of leukocytoclastic vasculitis

Noninflammatory vessel wall abnormalities	Disorders of collagen production and increased capillary fragility: scurvy, Ehlers–Danlos syndrome, solar purpura, steroid purpura, amyloidosis and trauma
Inflammatory vessel wall abnormalities or damage to the vessel wall by intravascular thrombi or emboli	Non-LCV vasculitis (e.g., lymphocytic vasculitis) Disseminated intravascular coagulation (DIC) Thrombotic thrombocytopenic purpura (PTI) Emboli: cardiac myxoma, cholesterol emboli, septic and infectious emboli Pigmented purpuric dermatosis Gardner–Diamond syndrome
Coagulation, platelet and other intravascular abnormalities	Platelet dysfunction disorders (e.g., Von Willebrand disease, Glanzmann disease, Wiskott–Aldrich syndrome, Bernard–Soulier syndrome) Thrombocytopenia Clotting factor defects

### Management

**Fig. 4:**
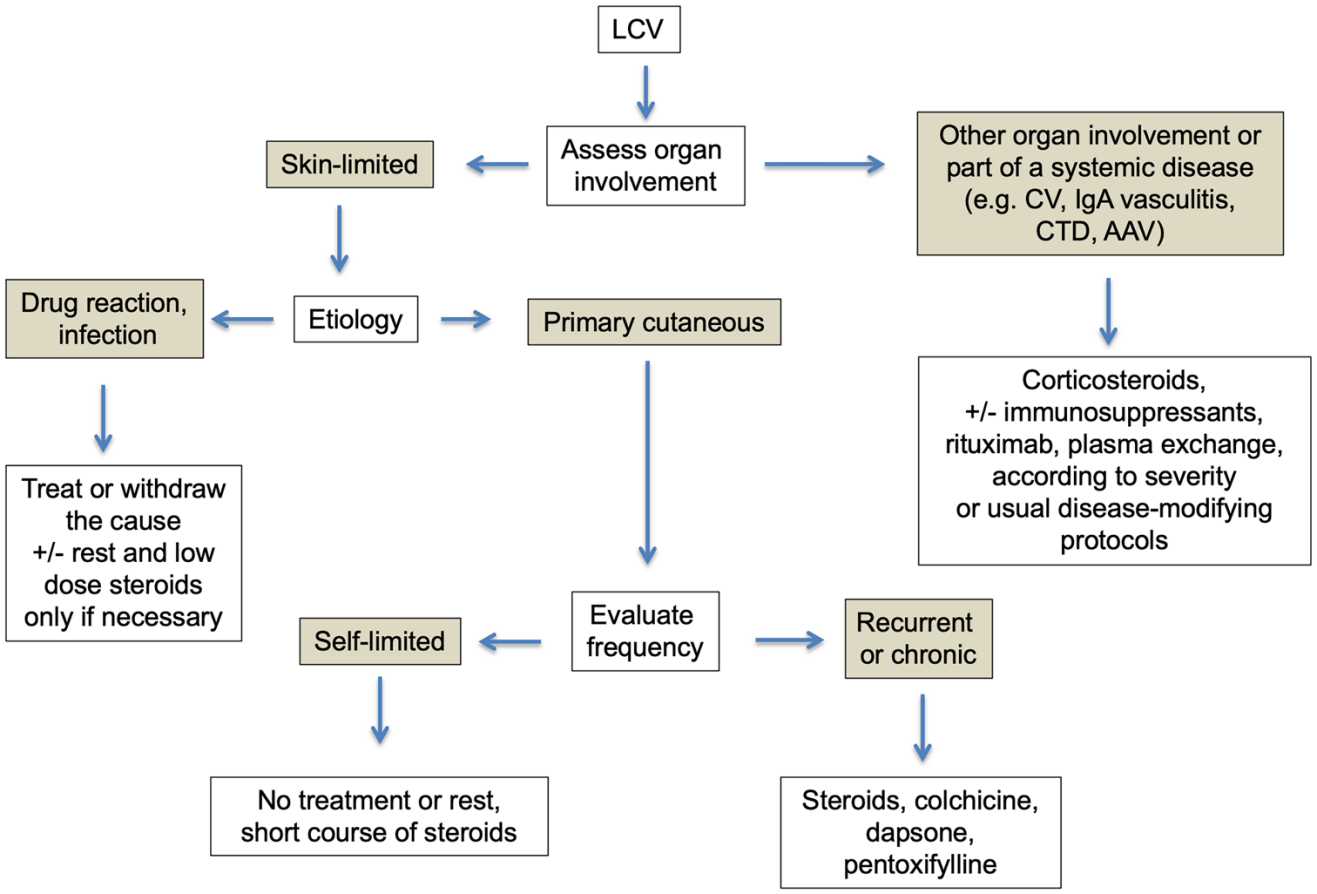
Approach to the treatment of leukocytoclastic vasculitis The treatment of LCV depends on etiology and the extent of organ involvement. Abbreviations: *CV* cryoglobulinemic vasculitis, *CTD* connective tissue disease, *AAV* ANCA-associated vasculitis

Once the diagnosis has been made, the treatment of LCV depends on two major factors: the etiology and the extent of disease (Fig. 4).

If LCV is limited to the skin, the management strategy should mostly focus on symptomatic relief, since the majority of acute episodes of cutaneous SVV are self-limited and do not recur, even without treatment [10]. Rest (avoiding prolonged standing or walking) and elevation and use of compression stockings should be advised in all cases.

When the cause of LCV is obvious, such as infections or drugs, eliminating or treating the trigger whenever possible is crucial and often sufficient. On the opposite, when a skin-limited SVV is severe, intractable or recurrent, the treatment should generally include systemic corticosteroids with or without adjunctive therapies.

Corticosteroids use is widely accepted but the dosage depends on severity; severe form may require initial doses of 0.5–1 mg/kg/day of prednisone equivalents. The response to steroid therapy is usually rapid, but the dose should be tapered slowly to prevent rebound. Long-term therapy may not be necessary if the process is self-limiting, but alternative options should be considered whenever necessary due to the well-known adverse events of corticosteroids.

Alternative options for the long-term management of skin-limited SVV include colchicine (0.5–1 mg per day, if tolerated). Colchicine improved skin and joint symptoms in open label studies, inducing prompt resolution of cutaneous vasculitis [44]. The use of colchicine may be limited by gastrointestinal side effects, mostly diarrhea.

Dapsone (50–200 mg/day) has also been reported to be effective in a small case series [45]. It is contraindicated in patients with glucose-6-phosphate dehydrogenase deficiency, as it can cause methemoglobinemia and hemolytic anemia, thus necessitating regular laboratory monitoring.

Finally, hydroxychloroquine (200–400 mg/day) may be beneficial in urticarial vasculitis [46], and NSAIDs may help to alleviate symptoms. The use of these agents is supported only by case series and anecdotal data.

When LCV occurs in the context of a systemic vasculitis or an underlying disease, or if none of the above-mentioned agents is effective or tolerated, immunosuppressive medications, such as azathioprine (1–2 mg/kg/day, if thiopurine methyltransferase levels are normal) [47], methotrexate (0.2–0.3 mg/kg/week) [48], with folic acid supplementation, and mycophenolate mofetil (2–3 g/day) [49] can be considered, balancing risks and benefits. Stronger and more toxic agents, such as cyclophosphamide may be effective, but should be limited to severe systemic vasculitides with organ-threatening involvement.

In general, the treatment depends on the diagnosis and aims to induce and maintain disease remission.

Treatment of HSP relies primarily on corticosteroids, although their use is debated in children. In previous placebo-controlled studies, steroids appeared to be effective for joint, intestinal and renal manifestations (such as proteinuria or haematuria), but not for preventing late-onset renal involvement or evolution to ESRD [50].

Glucocorticoids are frequently employed to reduce inflammation and immune complex formation in HUV. In patients with relapsing and/or refractory disease, rates of cutaneous and immunologic response to therapy seemed to be higher with conventional immunosuppressive agents, such as azathioprine, mycophenolate mofetil and cyclophosphamide [51].

The therapeutic management of CV depends on the underlying trigger and the severity of disease. When polyneuropathy or kidney involvement are present, immunosuppressive treatment or plasma-exchange strategies are usually required [52].

Finally, in the current era of biologic therapies, targeting anti-CD20 with rituximab has now an established role in the treatment of ANCA-associated vasculitides [53], as well as in cryoglobulinemic vasculitis [54] and HUV [51]. Rituximab appears promising also for HSP, when severe systemic or renal involvement favors an immunosuppressive or a corticosteroids sparing therapy [55].

### Prognosis

Most episodes of single-organ cutaneous LCV are self-limited, resolve over 3–4 weeks, with or without residual hyperpigmentation, and do not recur [10], [12], [56]. Overall survival is good (99 and 83% at 1 and 3 years, respectively), even in the case of recurrent LCV. Relapses may occur in less than 20% of the cases, especially when the cutaneous biopsy shows vascular thrombosis, in patients with peripheral neuropathy or hepatitis [10], in patients with extensive skin involvement [11].

In systemic forms, the prognosis largely depends on the severity of organ involvement as well as the extent of underlying disorder.

## Conclusion

LCV is a histopathologic term that defines vasculitis of the small vessels in which the inflammatory infiltrate is composed of neutrophils with leukocytoclasia phenomenon. LCV is not a single and specific disease but only a histopathologic definition common to several diseases. The prevalent cutaneous involvement has made the term of LCV synonymous of cutaneous vasculitis, or small vessel cutaneous vasculitis, often used interchangeably, generating confusion. The diagnostic approach to LCV almost invariably requires a skin biopsy and should be focused to understand if it is skin-limited or systemic, because treatment is consequently different. In limited forms, eliminating the cause and maintaining rest or low dose steroids are often sufficient, whereas in systemic vasculitis therapy is based on corticosteroids, immunosuppressive agents, rituximab or plasma exchange according to the extent and severity of the disease.
